# Noninvasive Diabetes Detection through Human Breath Using TinyML-Powered E-Nose

**DOI:** 10.3390/s24041294

**Published:** 2024-02-17

**Authors:** Alberto Gudiño-Ochoa, Julio Alberto García-Rodríguez, Raquel Ochoa-Ornelas, Jorge Ivan Cuevas-Chávez, Daniel Alejandro Sánchez-Arias

**Affiliations:** 1 Electronics Department, Tecnológico Nacional de México/Instituto Tecnológico de Ciudad Guzmán, Ciudad Guzmán 49100, Mexico; m21290934@cdguzman.tecnm.mx (A.G.-O.); m22290910@cdguzman.tecnm.mx (J.I.C.-C.); m21290935@cdguzman.tecnm.mx (D.A.S.-A.); 2 Centro Universitario del Sur, Departamento de Ciencias Computacionales e Innovación Tecnológica, Universidad de Guadalajara, Ciudad Guzmán 49000, Mexico; 3 Systems and Computation Department, Tecnológico Nacional de México/Instituto Tecnológico de Ciudad Guzmán, Ciudad Guzmán 49100, Mexico; raquel.oo@cdguzman.tecnm.mx

**Keywords:** electronic nose, diabetes mellitus, TinyML, exhaled-breath analysis, VOCs, TensorFlowLite

## Abstract

Volatile organic compounds (VOCs) in exhaled human breath serve as pivotal biomarkers for disease identification and medical diagnostics. In the context of diabetes mellitus, the noninvasive detection of acetone, a primary biomarker using electronic noses (e-noses), has gained significant attention. However, employing e-noses requires pre-trained algorithms for precise diabetes detection, often requiring a computer with a programming environment to classify newly acquired data. This study focuses on the development of an embedded system integrating Tiny Machine Learning (TinyML) and an e-nose equipped with Metal Oxide Semiconductor (MOS) sensors for real-time diabetes detection. The study encompassed 44 individuals, comprising 22 healthy individuals and 22 diagnosed with various types of diabetes mellitus. Test results highlight the XGBoost Machine Learning algorithm’s achievement of 95% detection accuracy. Additionally, the integration of deep learning algorithms, particularly deep neural networks (DNNs) and one-dimensional convolutional neural network (1D-CNN), yielded a detection efficacy of 94.44%. These outcomes underscore the potency of combining e-noses with TinyML in embedded systems, offering a noninvasive approach for diabetes mellitus detection.

## 1. Introduction

Diabetes mellitus represents a prevalent chronic disease demanding continuous monitoring and control due to its costly complications [1,2]. Neglecting proper care can diminish life expectancy and lead to severe complications such as nervous system damage, strokes, heart attacks, vision and kidney issues, and an increased risk of premature death [3,4,5,6,7]. This condition arises from either insufficient insulin production or the body’s ineffective use of it. Globally, over 530 million people live with diabetes, with projections indicating a staggering increase to 783 million by 2045. This surge is expected to drive healthcare expenses from USD 966 billion in 2021 to an estimated USD 1.054 trillion by 2045 [8,9].

While type 2 diabetes (T2DM) constitutes 90% of cases and can be managed with lifestyle changes like increased physical activity, reduced smoking, and timely diagnosis [10], type 1 diabetes (T1DM) lacks preventive measures and requires the continual monitoring of blood glucose levels (BGLs) alongside proper insulin administration [11,12]. Early diagnosis remains pivotal in managing diabetes. However, traditional detection methods, involving invasive blood extractions for BGL measurement, present drawbacks such as pain, ongoing strip costs, and disease transmission risks like hepatitis and human immunodeficiency virus (HIV) [13,14,15]. Consequently, the exploration of noninvasive techniques for diabetes detection, such as tear, saliva, urine, and breath analysis, has gained attention, although with limitations like inaccuracies in measuring minimal BGL, high sensitivity, and low detection limits [16,17,18,19,20].

**Figure 1:**
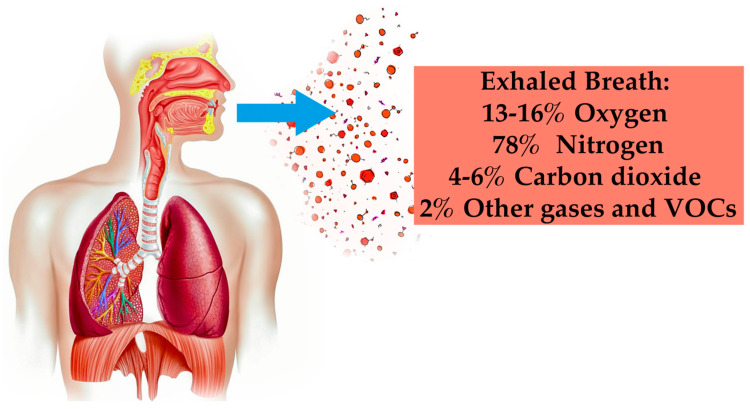
Components in exhaled human breath.

Exhaled human breath analysis emerges as a promising noninvasive method with potential for medical diagnostics. Human breath contains VOCs, serving as biomarkers for various diseases and enabling monitoring, prediction, prognosis, and risk assessment [21,22,23,24]. Some breath components indicating diseases are depicted in Figure 1. Identifying these biomarkers involves analyzing VOCs’ composition in healthy and diseased individuals. Notably, pulmonary biomarkers aid in understanding respiratory system-related processes and changes [25]. Clinical research on breath biomarkers has predominantly focused on diseases like lung cancers and asthma [26,27], cystic fibrosis [28], tuberculosis diagnosis [29], myocardial infarction [30], colorectal cancer [31], head and neck cancer [32], analysis in respiratory failure patients [33], gastric cancer detection [34], and importantly, metabolic disorders like diabetes [16,35,36,37,38].

Acetone, identified as a biomarker for diabetes in exhaled breath, presents lower concentrations in healthy individuals than in those with diabetes, supporting the estimation of BGL by correlating this biomarker with BGL ranges linked to elevated blood ketone [35,36,38,39,40]. Variability among patients is significant, as pathological changes alter breath composition, rendering human breath unique to everyone, akin to a fingerprint [27,35]. Additionally, the relative humidity (RH) and temperature of exhaled breath vary between 41.9% and 97% and between 31.4 °C and 34.8 °C, respectively [35].

Implementing advanced systems for breath analysis and diabetes, such as GC/MS (gas chromatography–mass spectrometry) [41], SIFT-MS (selected ion flow tube mass spectrometry) [42], and PTR-MS (proton transfer reaction–mass spectrometry) for detecting VOCs [43], proves expensive and demands specialized analysis [16,35].

Conversely, portable e-noses garner interest due to their noninvasive nature and swift response time in medical diagnosis; these devices can analyze breath components [16,23,24,35,37]. The use of integrated gas sensors, particularly those in the MQ series, has been widely reported in the literature, demonstrating reliability in detecting diabetes mellitus through exhaled human breath analysis [23,35,44,45,46]. Previous clinical studies in diabetes mellitus employed algorithms based on VOC features selection and extraction using Principal Component Analysis (PCA) [38,44,45,46,47,48]. Regression models in breath analysis provide quantitative BGL results with 90.4% accuracy [49].

Furthermore, qualitative prediction of diabetes has been validated using classical algorithms like Support Vector Machines (SVMs), K-nearest neighbors (KNN), and artificial neural networks [16,44,50]. Advanced machine learning and deep learning algorithms reveal superior accuracy in classifying healthy and diabetic patients, as well as among healthy, T1DM, and T2DM patients [16,35,38]. One-dimensional CNNs combined with SVM for feature extraction achieve 98% accuracy [51,52], while DNNs reach 96.29% accuracy in multilevel diabetes classification [44]. Decision tree-based algorithms like Random Forest [48] and XGBoost demonstrate 99% accuracy in artificial breath analysis, effectively avoiding overfitting issues [50].

Hence, utilizing e-noses for exhaled human breath analysis and integrating machine learning for diabetes detection present certain limitations [35]. Integrating new breath samples into a trained algorithm requires a computer, analyzing differences between samples from sick and healthy individuals [44,51,52]. However, there is a solution to this limitation using TinyML, offering edge artificial intelligence (AI) capable of processing data locally, without the need for data transfer, preserving information, reducing latency, energy consumption, and enhancing data privacy/security [53,54,55,56].

This approach meets all the requirements of cutting-edge AI computing and enables the efficient processing and classification of data quality [57,58], independent of resource-consuming cloud services or a computer for classifying new VOC-collected data [59]. In human breath analysis, the integration of TinyML and sensors as a noninvasive technique has been effective in predicting respiratory diseases such as chronic obstructive pulmonary disease (COPD) [60]. Moreover, it has shown success in the prediction of the BGLs of patients with T1DM with a CGM sensor and a recurrent neural network that builds on long-short term memory (LSTM) [61]. This tool has proven effectiveness in the broader health domain [62], facilitating predictions of vital metrics such as blood pressure, cough detection, the pre-screening of oral tongue lesions, and employing a head imaging system for brain stroke detection [63,64,65,66].

This research offers an application opportunity in medical diagnosis, explores a noninvasive approach, and offers a rapid, secure, and painless diagnostic alternative. It proposes integrating TinyML and Edge AI into an embedded system with an e-nose, combining advanced machine learning algorithms with deep neural networks for real-time detection and classification between healthy patients and patients with diabetes mellitus through exhaled human breath. The study aims to develop and implement an easily accessible device based on breath analysis, secure, and offering real-time early diagnosis. These contributions offer real-time classification using TinyML, resulting in low energy consumption and enabling machine learning applications through an e-nose for medical diagnosis without the need for a computer to process and classify new VOCs data from tests in healthy and sick patients. This can be implemented with any microcontroller compatible with TensorFlow Lite technology, validated through tests with healthy patients, patients with T1DM, and patients with T2DM.

## 2. Materials and Methods

The proposed e-nose system was developed for the analysis of breath samples obtained from two distinct groups: healthy individuals (HIs) and individuals diagnosed with diabetes mellitus (DMI) with T1DM or T2DM. To ensure the reliability of our measurements, the measurement system employed various preprocessing and normalization techniques aimed at mitigating atypical sensor noise. This section outlines the essential components of the e-nose, encompassing the selected gas sensors, the microcontroller, and a comprehensive overview of the system’s functionality. Furthermore, it provides an in-depth description of the calibration process specifically conducted for the MOS sensors, explains the breath sample collection procedure, and furnishes pertinent physical information about the study participants. The techniques utilized for preprocessing measurements and feature selection are also meticulously detailed.

### 2.1. E-Nose and Gas Sensors

**Figure 2:**
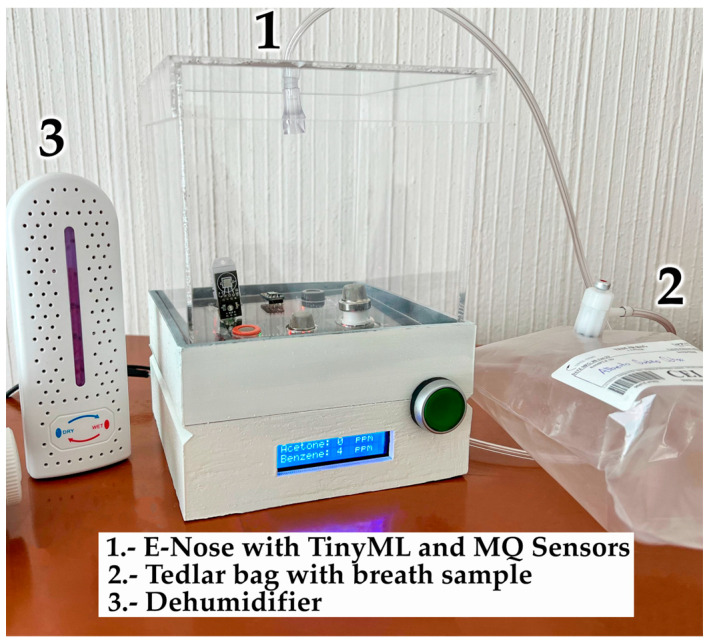
E-nose, dehumidifier, and Tedlar bag for breath samples.

The e-nose utilized in this study is depicted in Figure 2, featuring an array of 6 sensors housed within an acrylic sample chamber measuring 15 cm × 15 cm × 14 cm or 15 × 15 × 14 cm^3^ with a capacity of 3.5 L. Exhaled-breath samples from the participants were collected using Tedlar sample bags, each with a volume of 1 L. This widely acknowledged method of sample collection is commonly employed in breath sample analysis studies [35,45,46,47,48].

Given the primary focus of this study on identifying acetones as a marker for detecting DMI, the e-nose integrated catalytic gas sensors or MOS sensors from the MQ series (Waveshare, Shenzhen, China). These sensors are capable of detecting compounds such as carbon monoxide, alcohol, ketones, VOCs, temperature, and RH. The selection criteria for the implemented sensors are based on similar research for the detection of diabetes mellitus from exhaled breath [16,35,44,45,46]. Table 1 presents the sensors incorporated into the proposed system along with their respective gas detection ranges.

To monitor humidity levels within the sample chamber, a DHT-22 sensor was included due to the MOS sensor’s sensitivity to high RH present in exhaled-breath samples, which could potentially impact measurement outcomes. The high RH of exhaled air affects the results of the measurements, especially those performed with the use of MOS sensors [16,35]. To address this concern, a portable source 24V DC, 25-watt dehumidifier was integrated into the system. This dehumidifier effectively reduced RH levels within the e-nose sample chamber before and after each measurement session, ensuring a stable environment for accurate sensor readings throughout a 3-min duration.

**Figure 3:**
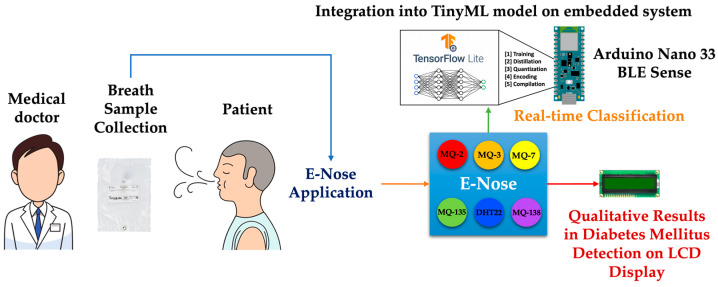
Scheme of the proposed TinyML-powered e-nose measurement system.

The e-nose system utilized in our experiment incorporates the Arduino Nano 33 BLE Sense due to its compatibility with integrating deep learning models using TensorFlow Lite and TinyML. Its standout features include a 12-bit ADC resolution and a 32-bit ARM Cortex-M4 processor. The implementation of TinyML allows for the direct execution of machine learning models on the device [53], thereby optimizing performance and efficiency in diabetes mellitus detection through the e-nose, as illustrated in Figure 3. Additionally, an LCD screen was included via the I2C communication bus for displaying temperature, RH, and relevant gas detection in parts per million (ppm). Before integrating the TensorFlow model into the microcontroller, the process involves breath sample collection through a serial communication protocol using Python version 3.11. The collected measurements from each patient’s tests were stored in an Excel (.csv) file. The calibration of the gas sensors is detailed in Section 2.3 before preprocessing and feature selection are discussed.

### 2.2. Gas-Sensing Mechanism

The gas-sensing mechanism of each sensor utilized in this study is described below:

- Gas sensors of the MQ series: The sensing mechanism is based on the principle of detecting changes in the electrical conductivity of a semiconductor material when exposed to specific gases. These sensors consist of a thin layer of metal oxide deposited on a ceramic or glass substrate. Upon interaction with gases, chemical reactions occur on the surface of the semiconductor material, initiating with the adsorption of gas molecules. This process alters the electronic structure of the metal oxide layer, resulting in a change in its electrical conductivity. The magnitude of this conductivity change is directly proportional to the concentration of the target gas in the environment, facilitating the determination of gas presence and concentration [16,24,35,37].
- MICS-5524 sensor: The gas-sensing mechanism relies on an electrochemical detection principle to measure the concentration of specific gases in the environment. This sensor is constructed with a catalytic detection electrode sensitive to the target gas and a counter electrode. Upon coming into contact with the target gas, a chemical reaction occurs at the detection electrode, generating an electric current proportional to the gas concentration. This electric current is then amplified and converted into an output signal that can be measured and quantified to determine the concentration of the target gas. This process enables the precise and sensitive detection of gases across a wide range of applications [16,23,24].
- DHT-22 sensor: The sensor operates based on capacitive humidity sensing coupled with a thermistor for temperature measurement. This sensor comprises a humidity-sensitive capacitive element and a thermistor enclosed within its housing. The capacitive element detects changes in electrical capacitance caused by variations in the surrounding air’s moisture content, facilitating precise humidity measurements. Simultaneously, the thermistor measures temperature variations by detecting changes in its electrical resistance. Both humidity and temperature data are then processed and outputted by the sensor, providing accurate and reliable environmental measurements. This combination of capacitive humidity sensing and thermistor-based temperature measurement ensures the effectiveness of the DHT-22 sensor in various applications requiring precise monitoring of humidity and temperature levels [67,68].

### 2.3. Sensor Calibration and Patient Breath Sample Collection

**Figure 4:**
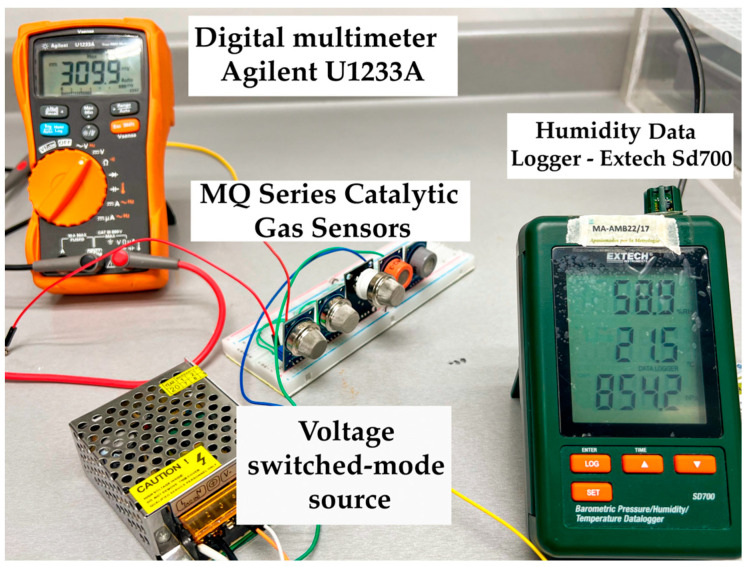
Calibration procedure for MQ gas sensors in a space with optimal RH conditions.

Due to the sensitivity of the MQ gas sensors, calibration was conducted under varying RH conditions and clean air ranges to obtain the characteristic gas detection curve, excluding the DHT-22 and MICS-5524 sensors, as their curves are provided in their respective datasheets. Notably, MQ sensors require a maximum preheating time of 48 h using a switched voltage source before initial use. The calibration procedure was executed at MetAs S.A. de C.V. laboratories in Ciudad Guzmán, Jalisco, Mexico. The essential instruments employed for calibration included a precision 4.5-digit Agilent U1233A digital multimeter and an Extech Sd700 Datalogger for temperature and RH measurement, as depicted in Figure 4.

The environmental conditions were maintained at 55–65% RH and in a temperature range of 20–22 °C, crucial for establishing the sensitivity characteristic relationship between sensor resistance, Ro, and measured resistance, Rs, as shown in Equation (1) below.

(1) $$\frac{\mathrm{Rs}}{\mathrm{Rl}}=\frac{V-\mathrm{Vs}}{\mathrm{Vs}}$$

Here, V represents the supplied voltage to the sensor, and Vs is the current sensor reading in volts, while Rl corresponds to the fixed load resistance. Equation (2) signifies the Ro value when the sensor operates in clean-air conditions:

(2) $$\mathrm{Ro}=\frac{\mathrm{Rs}}{\mathrm{Clean}\;\mathrm{Ratio}}$$

Using a programmed routine involving regression and extraction of target gas curve points for each sensor, the respective potential equation is derived. After sensor calibration, breath samples were collected and measured from a total of 44 participants. Among them, 22 were HI, 8 were individuals with T1DM (T1DMI), and 14 were individuals with T2DM (T2DMI). All participants provided their consent to participate in the experimental study by signing a consent form. The e-nose system was initialized five minutes in advance to preheat the internal sensor resistances and stabilize temperature and RH values within the sample chamber.

**Figure 5:**
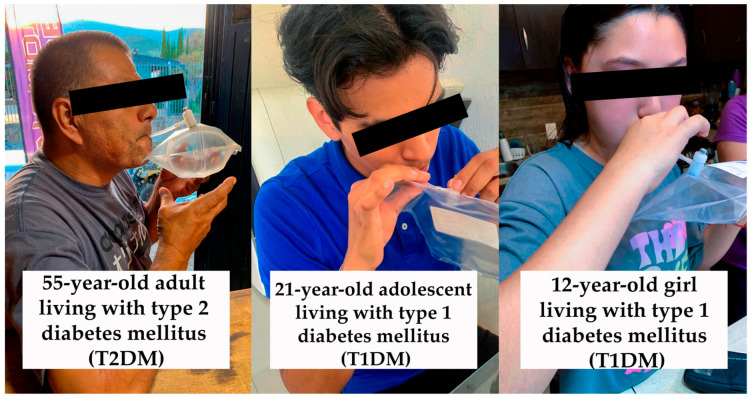
Patients with T2DM and T1DM in the exhaled-breath sample collection procedure.

The dataset includes sensor measurements for the respective target gases in ppm and Rs/Ro values, along with temperature and RH measurements. Participants were instructed to inhale to their maximum lung capacity and exhale completely through a respiratory mouthpiece connected to Tedlar bags, filling them to 90–100% capacity, as depicted in Figure 5. Additionally, their BGL was measured using a glucometer to confirm their status as either HI or DMI.

After breath collection, a waiting period of 5–10 min was observed before transferring the breath sample to the e-nose. This ensured a temperature reduction within the Tedlar bag. On average, across all participants, there was an average RH of 69.745% and a temperature of 32.905 °C. Measurements were taken for 90 s during the transfer of the breath sample from the Tedlar bag to the sample chamber. Using Python encoding and serial connection, 10,000 measurement values were acquired per patient per test.

Physical information about the recruited participants, including age, gender, Body Mass Index (BMI), among other parameters, is presented in Table 2.

### 2.4. Data Preprocessing and Feature Selection

After collecting the dataset for each patient, preprocessing was crucial to mitigate signal noise caused by the inherent variability in RH, breath temperature, and voltage fluctuations from the power source [35]. The Discrete Wavelet Transform (DWT) was applied in Python to achieve this goal. DWT decomposes the signal into components of different scales and frequencies, enabling the identification and elimination of noise [44]. Equation (3) illustrates the governing equation for DWT:

(3) $$\mathrm{DWT}\left(f,\;a,\;b\right)=\frac{1}{\sqrt{a}}\int_{\infty }^{\infty }f\left(t\right)\psi \left(\frac{t\;-\;b}{a}\right)\mathrm{dt}$$

where f is the original signal, ψ represents the mother wavelet function, and a and b are scale and translation parameters, respectively. In the context of data processing, signal filtering was performed using DWT to remove noise. The “VisuShrink” thresholding method was employed to reduce wavelet transform coefficients. This involved visually inspecting coefficients and deciding which to retain or discard. Equation (4) illustrates the key thresholding criterion:

(4) $${\mathrm{Shrinkage}}_{\mathrm{Visu}}\left(x,\lambda \right)=\mathrm{sign}(x){(\left|x\right|-\lambda )}_{+}$$

where x represents wavelet coefficients, λ is the threshold, sign(·) is the sign function, $\left|x\right|$ is the magnitude of coefficients, and ${\left(\left|x\right|-\lambda \right)}_{+}$ is the soft threshold, setting values below λ to zero. The “soft” thresholding mode was chosen to achieve a smooth thresholding effect. Equation (5) demonstrates this method by eliminating coefficients below the threshold and proportionally reducing others:

(5) $${\mathrm{Shrinkage}}_{\mathrm{Soft}}\left(x,\lambda \right)=\mathrm{sign}(x)\cdot \max(\left|x\right|-\lambda ,0)$$

The decomposition level was determined based on Equation (6), which equaled 1, a significant value balancing signal integrity and noise sensitivity. The “db6” Daubechies 6 wavelet was chosen for its signal compression and noise elimination nature.

(6) $$\frac{F_{q}}{2^{L+1}}\leq F_{\mathrm{char}}\leq \frac{F_{q}}{2^{L}}$$

where $F_{q}$ represents the sampling frequency, $F_{char}$ denotes the dominant frequency, and L signifies the decomposition level. Subsequently, feature scaling was conducted using Z-score normalization for CO, alcohol, acetone, ketones, humidity, and temperature sensor readings; this process ensured all signals were standardized onto a similar scale, exhibiting properties akin to a normal distribution, thereby facilitating comparison and analysis within the study. Equation (7) demonstrates Z-score normalization, also known as standard normalization [35,44,49].

(7) $$Z=\frac{X\;-\;\mu }{\sigma }$$

where X represents the original sample value, μ is the sample mean, and σ is the sample standard deviation. After noise removal and the normalization of the signal, the average value of each sensor in the individual participant’s test was obtained.

**Figure 6:**
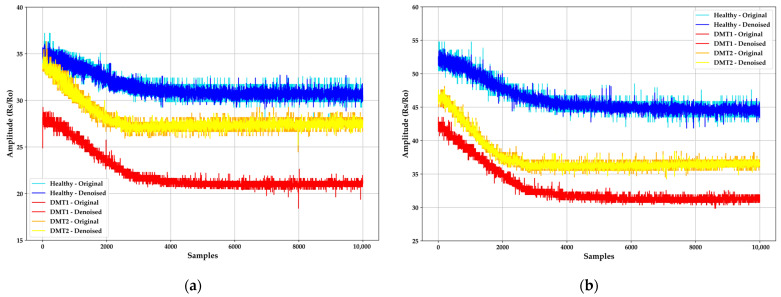
Response of Rs/Ro signals from the e-nose before and after noise elimination with DWT. (**a**) MQ-135 sensor response in the presence of acetones; (**b**) MQ-2 sensor response in the presence of carbon monoxide.

Figure 6 displays the Rs/Ro signals acquired from the MQ-135 and MQ-2 MOS sensors, illustrating the variability in sensor sensitivity concerning BGL for HI (92.5 mg/dL), T1DMI (139.28 mg/dL), and T2DMI (180.10 mg/dL). The amplitude is higher for HI due to the resistance relationship and lower ketone presence, aligning with the characteristic curve provided in the datasheet. As the ppm concentration increases, the Rs/Ro ratio decreases. In the case of the MQ-2, similar behavior is observed between T1DMI and T2DMI regarding Rs/Ro ratios, while HI patients exhibit minimal carbon monoxide concentration.

Focusing on the Rs/Ro values from the MQ sensors was chosen due to noticeable differences being observed among the measurements. Additionally, given the minimal variations in ppm levels between participants within both the HI group and the DMI group, emphasis was placed on the sensor voltage values from the MICS-5524 and the RH and temperature data from the DHT-22 sensor.

A statistical method based on feature selection was applied to retain the most informative data, even though our e-nose sensor matrix comprises only seven characteristics or measurement parameters [35]. This differs from other e-noses containing responses from ten different gas sensors [48]. Nevertheless, reducing the number of characteristics enhances the accuracy of machine learning and deep learning models. This optimization also speeds up training and reduces computational complexity, helping to prevent overfitting [35,36,47].

**Figure 7:**
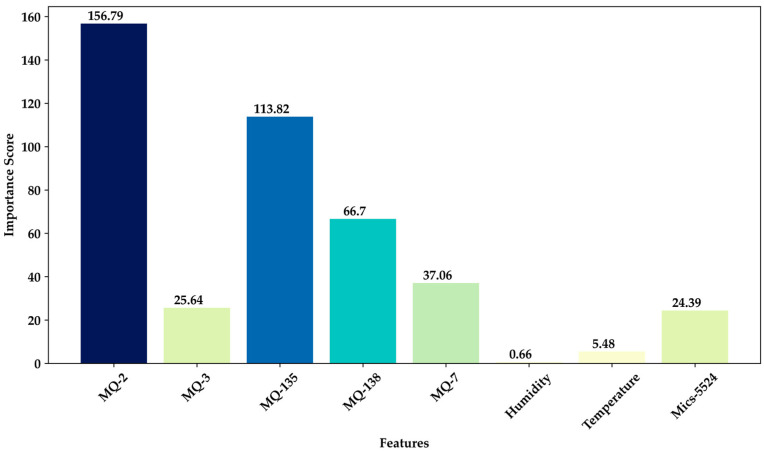
Importance scores of selected features from the breath sample dataset.

The univariate feature selection algorithm was implemented to refine the sensor features, aiming to enhance model precision while mitigating the risk of overfitting. This method evaluates the statistical relevance of each feature using a score function. The parameter “best features” was set to 4, indicating the desired number of features for selection. Subsequently, by extracting the indices of the selected features, specific sensor attributes that significantly contribute to the classification process were identified. Figure 7 illustrates the importance scores of the selected characteristics from each sensor. Notably, the measurements of acetone by the MQ-135 sensor and carbon monoxide by the MQ-2 sensor exhibited significant contributions. Consequently, only the top 4 selected characteristics from this process, including carbon monoxide, alcohol, acetone, and benzene, were utilized in the machine learning and deep learning models before training [44,46]. This feature selection process efficiently reduced the number of sensor attributes while retaining the most informative ones [35], thereby enhancing the performance and computational efficiency of our e-nose-based models post-training and conversion into TensorFlow Lite for the embedded system [53,55].

**Figure 8:**
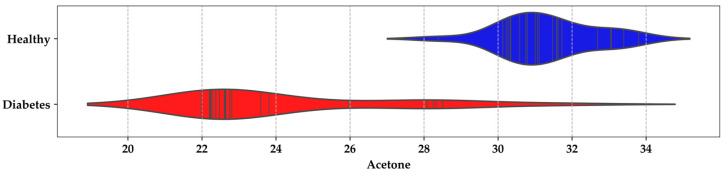
Violin plot for acetone concentrations in the breath of HI and DMI groups.

As part of the breath sample analysis, emphasis was placed on the acetone values in the Rs/Ro relationship between the HI and DMI groups; In Figure 8, a density range of 30–32, with a confidence interval of 28–30, was notable for the HI group, corresponding to BGL levels between 80.59 and 94.63 mg/dL. In contrast, for patients with T1DM or T2DM, the range was between 22 and 24, corresponding to BGL levels of 139.28–303.10 mg/dL. Based on measurements from the 44 participants, it was assumed that the exhaled-breath concentrations for an HI individual are <0.8 ppm, while for a DMI individual, they are ≥1.2 ppm.

**Figure 9:**
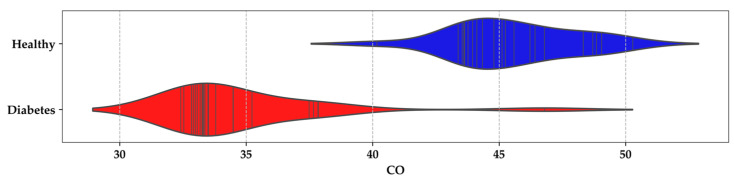
Violin plot for carbon monoxide concentrations in the breath of HI and DMI groups.

Furthermore, in Figure 9, a similar pattern is observed for the values of carbon monoxide in the exhaled breath among participant groups. A significant density range between 33 and 35 is noted for DMI, while a noticeable difference is observed for the HI group within the range of 44–47.

Before integrating the data into training the machine learning and deep learning models, PCA was utilized as an unsupervised learning technique solely for visualizing the optimal separability or classification of breath samples from each patient (this process does not form part of the TinyML integration) [16,35,46]. PCA ensures that the principal components remain uncorrelated. This algorithm identifies the axis that retains the highest variance within the training dataset, producing a set of mutually orthogonal axes equal to the dimension of the features [45,47,48]. Equation (8) mathematically represents this process:

(8) $$X_{d}=X\;\cdot \;W_{d}$$

where $X_{d}$ represents the outcome of the dimensionality reduction, and d signifies the desired number of dimensions. X denotes the matrix of the original dataset, and $W_{d}$ represents a matrix comprising the first principal component values derived from the singular value decomposition (SVD) method.

**Figure 10:**
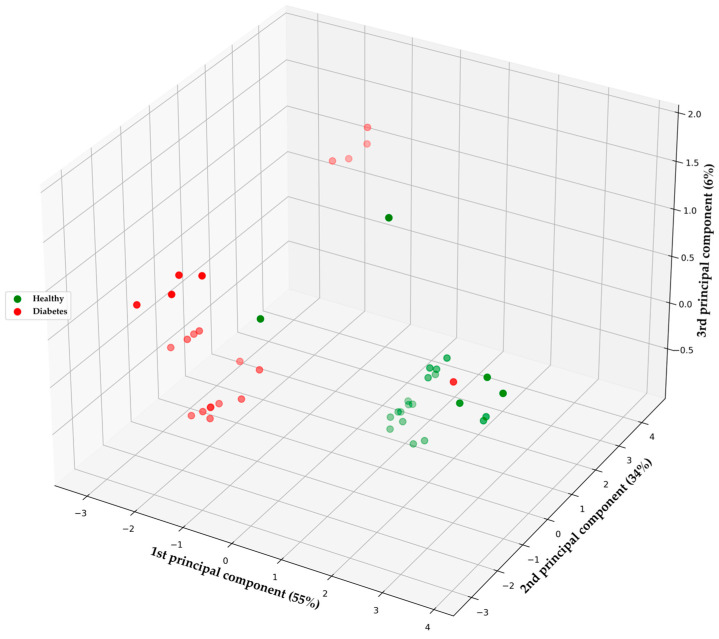
PCA visualization with scaling to assess breath samples among HI and DMI groups.

Figure 10 illustrates the clustering of breath samples from each patient, demonstrating the separability of the data based on the variance in BGL between HI and those diagnosed with T1DM or T2DM. In this case, the percentage of variance explained by PC1 corresponds to 55%, while PC2 accounts for 34%, and PC3 for 6%. Patients with diabetes exhibited higher BGL compared to the healthy group. This figure effectively demonstrates the distinction between these normal and high BGL groups and their direct correlation with the collected breath samples. Such a metric possesses the capability to differentiate between HI and DMI, emphasizing its pivotal role in developing machine learning and deep learning models that avoid overfitting for the classification of new data.

## 3. Results

This section focuses on the training and classification of models compatible with TinyML, as employed in this study. The models include an Extreme Gradient Boosting (XGBoost) machine learning model, a fully connected deep neural network (Dense NN), and 1D-CNN. Comparative results are presented across various classic machine learning models, with a focus on scoring metrics, particularly recall, F1-score (or specificity), and the Area Under the Receiver Operating Characteristics (ROC) curve (AUC-ROC). This integration serves as a prerequisite for implementation into an integrated system using TinyML, facilitating qualitative classification between HI and DMI groups [45,50]. Additionally, we conducted a comparison of classification times for newly exhaled-breath samples using the trained algorithms. Furthermore, the byte size of the models, converted into TensorFlow Lite and C++ language for the Arduino microcontroller [53], is detailed to achieve an embedded system.

### 3.1. Classification with XGBoost

The XGBoost algorithm has demonstrated remarkable effectiveness in analyzing human breath to identify acetone biomarkers, showcasing its viability due to its high efficiency in handling missing data [48,50]. Its performance benefits further from parallelization and hardware optimization, setting it apart from other machine learning algorithms by being less impacted by feature scale differences [35]. Given the limited number of participants in the dataset, a 55% split for training and a 45% split for testing were employed. Using the selected number of features, a binary logistic classification model was trained. Hyperparameter tuning was carried out through grid search and a 5-fold cross-validation method, consistently demonstrating performance in line with similar studies. The best model achieved an accuracy of 95%. Table 3 provides details on the optimal hyperparameters obtained after training the data.

**Figure 11:**
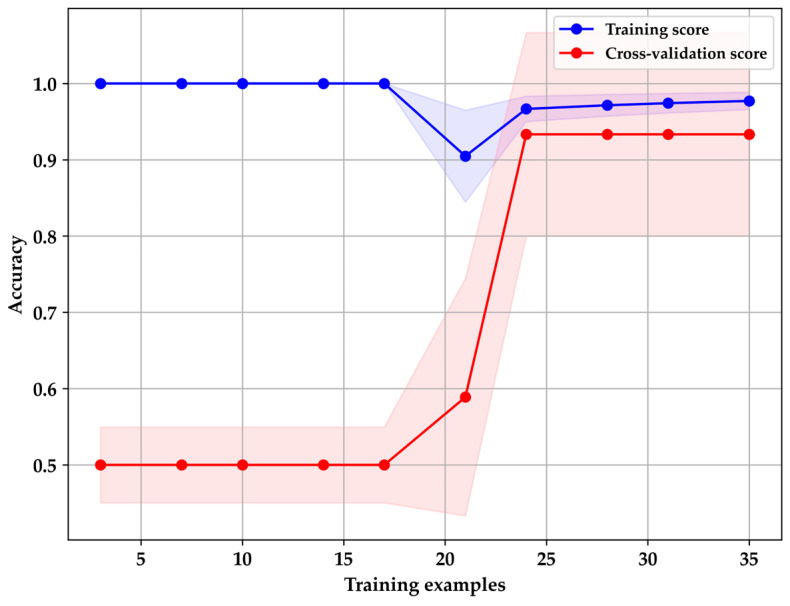
Learning curves generated using XGBoost.

Following hyperparameter tuning, a learning curve was generated, as depicted in Figure 11, derived from the mean and standard deviation of model accuracy scores across each training and cross-validation fold. The classification report in Table 4 underscores the importance of recall values, particularly in the classification of HI patients. It highlights the model’s ability to correctly identify individuals with diabetes, minimizing false negatives, a crucial aspect for an early and precise detection approach, enabling timely medical interventions [16,35,36,50].

In terms of precision, the algorithm proves effective in detecting DMI with accuracy in most cases, demonstrating an important recall of 90%. However, precision alone does not account for false negatives, indicating individuals with diabetes that the model fails to identify. Thus, while high precision ensures correct positive predictions, it may not be sufficient if the model has a high rate of false negatives, potentially leading to the omission of people with diabetes mellitus [44,45,46,47,48,49].

**Figure 12:**
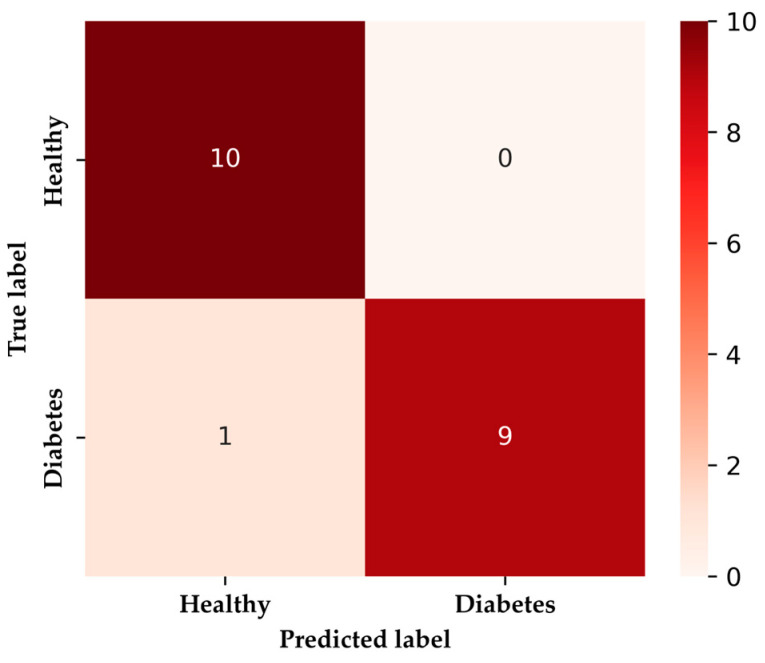
XGBoost confusion matrix.

Figure 12 visually illustrates instances where the model incorrectly predicts an individual as healthy (negative class) when they have diabetes (positive class), representing a type II error. Out of a total of 20 instances, the model correctly predicted 10 cases of HI and 9 cases of DMI.

### 3.2. Classification with Deep Neural Networks

The DNN model is structured on a sequential architecture, allowing for adaptability during conversion to a TensorFlow Lite file for subsequent deployment on the microcontroller. The dataset is divided, allocating 60% for training and 40% for testing. Furthermore, the training dataset is subdivided, with 60% representing the original data for training and an additional 25% for validation.

**Figure 13:**
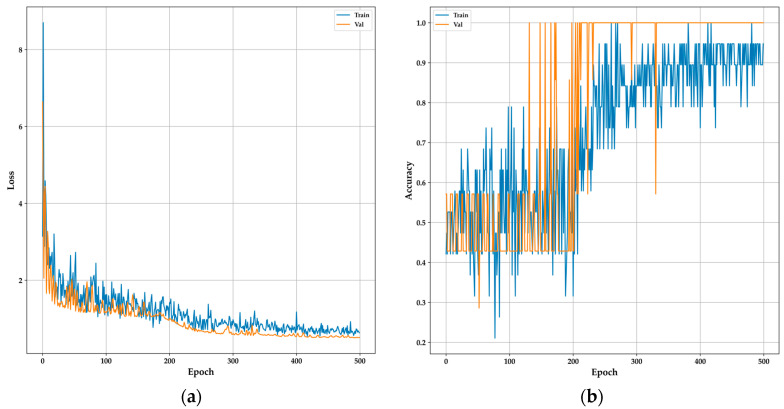
Performance of the DNN model during training: (**a**) model loss of DNN; (**b**) model accuracy of DNN.

The model parameters include a fully connected layer architecture within a sequential model. The input layer comprises 20 neurons with the “ReLu” activation function, followed by a hidden layer with 1200 neurons using the same activation function. The output layer consists of one neuron for binary classification, employing the sigmoid activation function. A dropout value of 0.1 was applied between the layers to enhance model robustness. The compilation involves the “adam” optimizer, which has exhibited superior effectiveness in classification, as demonstrated in similar studies [44], and binary crossentropy loss. The model underwent training over 500 epochs to achieve optimal classification. Figure 13 displays the loss and accuracy metrics throughout the training process, showing balanced behavior between validation and training data after 200 epochs to prevent overfitting. An impressive accuracy of 94.44% was achieved with only four selected features from the sensors, demonstrating acceptable performance.

**Figure 14:**
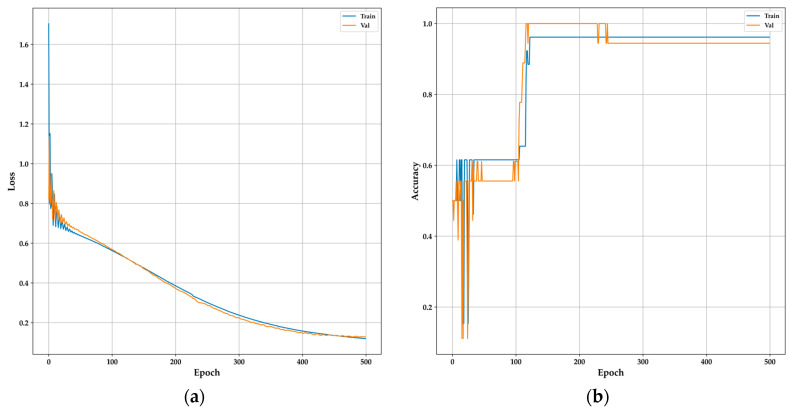
Performance of the 1D-CNN model during training: (**a**) Model loss of 1D-CNN; (**b**) model accuracy of 1D-CNN.

**Figure 15:**
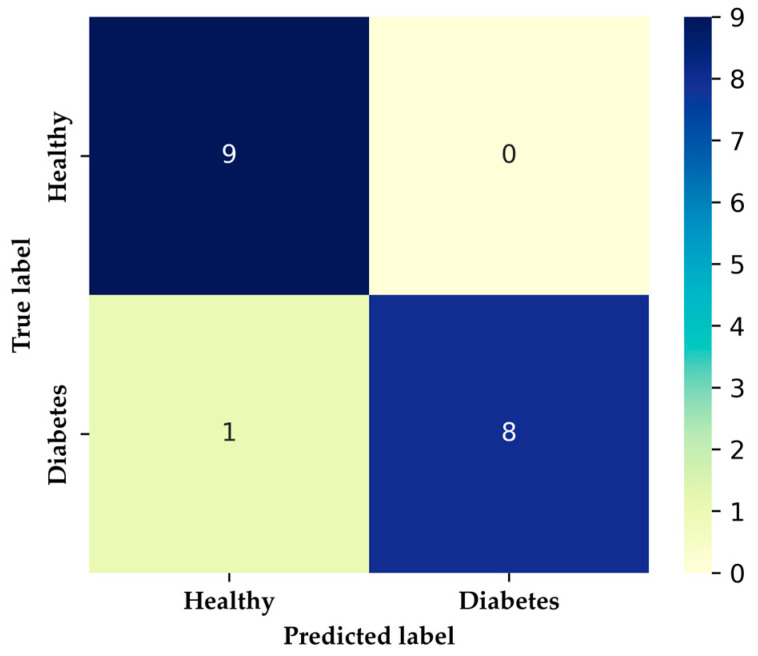
DNN and 1D-CNN confusion matrix.

The 1D-CNN model involves a split of the data, allocating 60% for training and the remainder for the test set. Data were reshaped for use by a one-dimensional convolutional layer, assuming the presence of four selected features. In this configuration, each instance comprises multiple features, and the one-dimensional convolutional layer takes one-dimensional windows of these features to extract relevant characteristics for classification [16,51,52]. The algorithm is based on a sequential model similar to DNN, with the convolutional layer set at 128 filters and a kernel of 3, utilizing the “ReLu” activation function. MaxPooling1D reduces the output dimensionality of the convolutional layer, converting the output into a one-dimensional vector. Two fully connected layers are included, one with 10 neurons and an output neuron with a sigmoid activation function. The compilation uses the same optimization parameters and loss function as the DNN model over 500 epochs. Consequently, the model learned to predict the binary variable HI or DMI using one-dimensional convolutions on the selected features. The results for both the DNN and 1D-CNN models are similar in scoring metrics, as presented in Table 5. Unlike the XGBoost model, the precision in HI patients was lower, while the recall in DMI patients was 89%. However, Figure 14 demonstrates improved performance with loss metrics consistently below 0.2 and non-overfitting accuracy after 200 epochs during training, compared to the DNN model. In comparison to the DNN model, the one-dimensional convolutional model has shown superior performance in similar studies on multiclass DMI groups classification [51,52]. The confusion matrix for both models is similar, as depicted in Figure 15, along with XGBoost, indicating the presence of a false negative for a patient classified as HI but is DMI. The model accurately identified 94.4% (9 out of 18) cases for HI and 8 for DMI in the test set.

### 3.3. Comparison with Classic Machine Learning Algorithms and Deep Neural Networks

The application of TensorFlow Lite or TinyML is not available for some machine learning models due to limitations in model conversion [52,53,54]. However, the performance of algorithms for detecting HI or DMI patients through new exhaled-breath tests was evaluated. Superior performance was observed in algorithms based on decision trees, such as XGBoost and DecisionTree [48,50]. Additionally, the implementation of Support Vector Machines (SVMs) with hyperparameter tuning, using grid search and cross-validation, including an RBF kernel and a gamma value of 0.1, showed metrics similar to XGBoost with 95% accuracy, 100% precision, and 90% recall. The F1-score was 95% for XGBoost and 94% for SVM.

**Figure 16:**
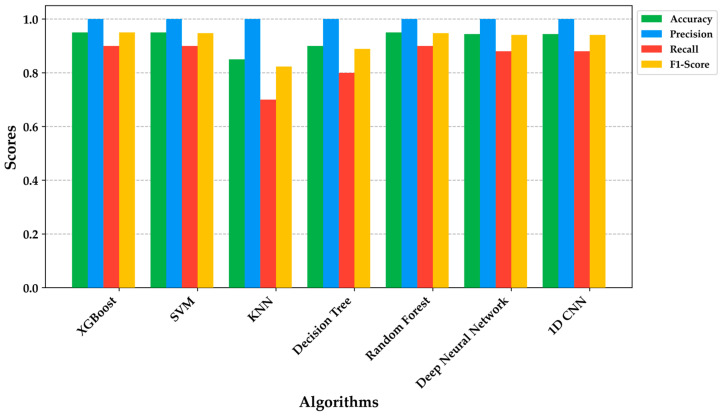
Accuracy, precision, recall, and F1-score comparison of different algorithms.

Comparison with other algorithms is presented in Figure 16, where classical machine learning and deep learning algorithms achieved a precision of 100%. Notably, XGBoost had the highest precision, followed by SVM, Random Forest, DNN, and 1D-CNN. Emphasizing recall, the worst performance was observed in KNN, while DNN and 1D-CNN had a recall of 88% [35]. XGBoost, SVM, and Random Forest demonstrated greater robustness in detecting HI and DMI patients. It is worth noting that the new tests were effective in detecting patients with T2DM who had higher BGL.

**Figure 17:**
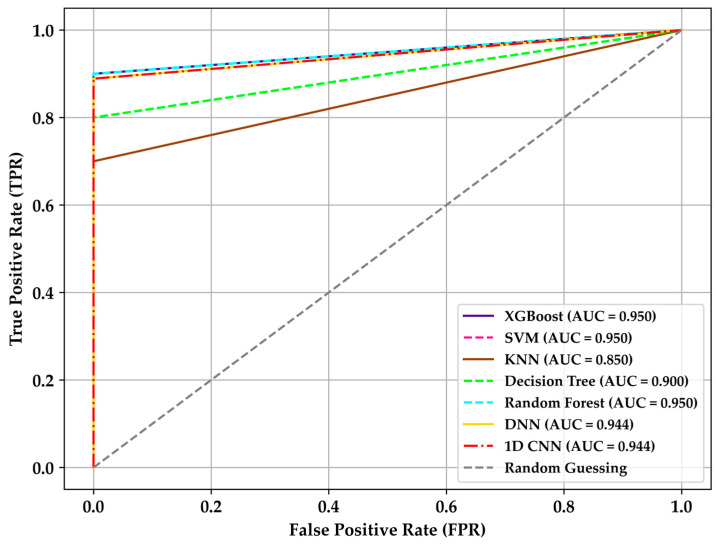
ROC curves comparison of different algorithms.

In Figure 17, the understanding of ROC curves clearly demonstrates the superiority of XGBoost, SVM, and Random Forest with a value of 95%, followed by deep learning-based models. For the dataset used, the inferiority of KNN and Random Forest in detecting DMI is noticeable, with values of 85% and 90%, approaching false positives. Detecting as many true positives as possible while reducing the number of false positives is crucial [16,35,36], as well as understanding the approach of this research in implementing TinyML with TensorFlow Lite on a microcontroller for qualitatively predicting the health status of patients with diabetes mellitus based on ketones related to their BGL, without the need for pre-processing data or the use of the Internet of Things or cloud services [53,54,55,56]. The most accurate algorithm is XGBoost due to its superior conversion features to a TensorFlow Lite model, detailed in Section 3.4. Although SVM and Random Forest are robust, they are not yet adapted to TinyML. On the other hand, DNN and 1D-CNN models, with 100% conversion features associated with TensorFlow, are viable for implementation in the embedded system as they rely on Keras and TensorFlow Lite libraries, presenting better conversion and acceptable performance [53].

### 3.4. Integration of TinyML into Arduino Nano 33 BLE Sense

While the XGBoost model has proven to be the most suitable algorithm for the qualitative detection of low BGL for HI or high levels for DMI, its ability to convert models from different libraries or algorithms to TensorFlow-compatible, and then to TensorFlow Lite models, depends on conversion compatibility [55]. This poses challenges for algorithms like SVM, KNN, Random Forest, and Decision Tree. However, XGBoost has specific functions and tools for exporting models, making it more compatible with conversion to TensorFlow and TensorFlow Lite. The process involves directly exporting the XGBoost model to TensorFlow, retraining the model with the Adam optimizer and binary cross-entropy loss, and finally converting it to a TensorFlow file, which is then saved and loaded onto an Arduino. However, a disadvantage of this approach is that implementations used by XGBoost and TensorFlow may differ slightly, leading to variations in predictions and consequently affecting performance metrics [56].

The accuracy decreased from 95% to 91.3%, while precision remained the same, recall changed from 90% to 80%, and the F1-score changed from 95% to 88.88%. Differences in metrics between the original and TensorFlow-converted models can be attributed to numerical precision variations between the two libraries, which might affect predictions. Such variations are common when converting models between different libraries or frameworks. Although acceptable if not significant, it is crucial to note that recall, an important metric for diabetes diagnosis and a supporting tool for medical doctors using the e-nose, can impact result credibility with false positives [16,35].

**Figure 18:**
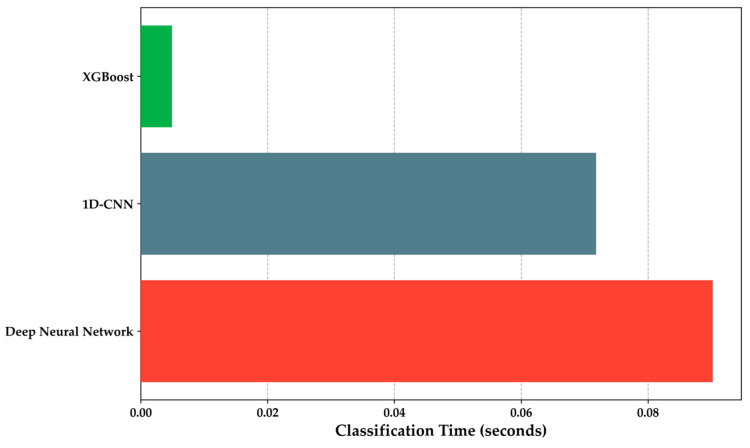
Comparison of classification time by algorithms in seconds.

For DNN and 1D-CNN models, conversion to TensorFlow Lite and loading onto the microcontroller occurred without issues, maintaining performance metrics for binary prediction between HI and DMI groups. Therefore, a performance metric comparison was conducted for classification time on new samples, predicting HI or DMI cases. Lower classification times are better for the microcontroller, considering available RAM for new data measurement, preprocessing, and classification. Figure 18 illustrates that the DNN model takes 0.09 s for new measurements, the 1D-CNN takes 0.01718 s, and notably, XGBoost demonstrates better performance in predicting new instances in 0.0049 s. However, the performance of this model is not the best among the three algorithms implemented for detecting diabetes mellitus.

**Figure 19:**
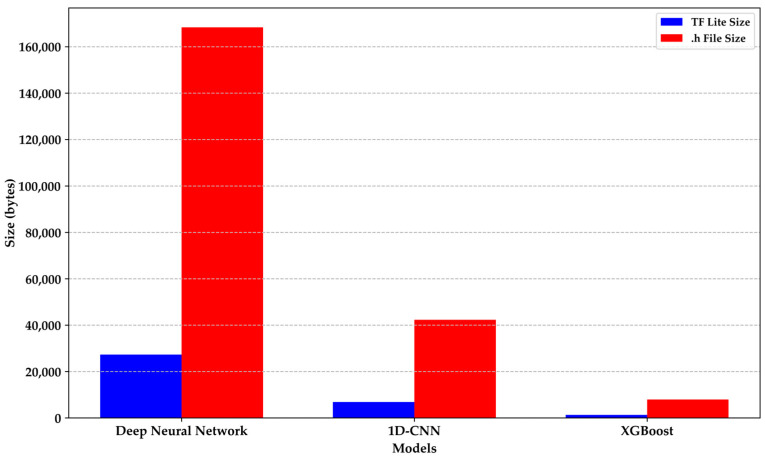
Sizes in bytes of the models in TensorFlow Lite and their conversion to .h files.

Regarding the importance of model size for integration into a microcontroller, it is undoubtedly crucial. The size of the resulting file, especially when converting it to a format like .h for the Arduino Nano 33 BLE Sense, is significant, given the storage and processing limitations of these devices [53,56]. Figure 19 illustrates the relationship between the size of the TF file and its conversion to the .h format before embedding it in the microcontroller. Once again, the XGBoost model demonstrates a smaller size compared to models based on deep learning. It is noteworthy that the e-nose application shows improved performance with the 1D-CNN model without significantly affecting the memory occupancy of the microcontroller when making new predictions for DMI or HI patients.

In the application of breath analysis in the embedded system of the e-nose, a significant outcome was obtained for distinguishing between patients with HI and DMI. Assuming four characteristics selected from MQ-2, MQ-3, MQ-135, and MQ-138 sensors, the DHT-22 was used solely to monitor the %RH of exhaled breath in the e-nose sample chamber. It is important to emphasize the importance of selecting these MQ gas sensors and their effectiveness in relation to diabetes mellitus detection, especially for carbon monoxide and acetone biomarkers in exhaled breath, as previously demonstrated [16,35,44,45,46].

**Figure 20:**
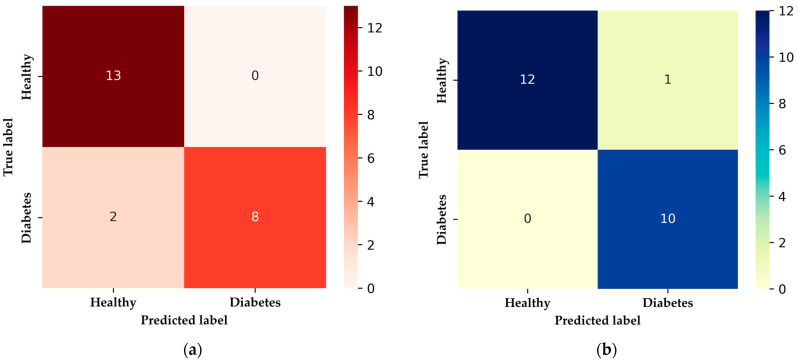
Confusion matrices of TinyML-implemented algorithms on microcontroller: (**a**) predictions with XGBoost algorithm; (**b**) predictions with DNN algorithm.

In Figure 20, the confusion matrix for the XGBoost algorithm illustrates its ability to correctly identify 13 healthy patients and 8 patients with diabetes mellitus, with two false negatives. On the other hand, the DNN model correctly detected 12 cases of HI and 10 of DMI, yielding a false positive, which is particularly relevant in medical applications. This scenario could potentially lead to individuals with T1DM or T2DM conditions and elevated BGL not being accurately identified. Only the XGBoost and DNN models were implemented, omitting the Conv1D-based 1D-CNN model due to the complexities associated with adapting the measurements for compatibility with Conv1D. It is noteworthy that the DNN model exhibited a false positive, underscoring the importance of carefully considering false positives in medical applications [16,35,36,45], where the consequences of misclassification can be significant. The decision to exclude the 1D-CNN model was made due to the challenges associated with reshaping measurements to fit the Conv1D model, highlighting a potential area for future research or optimization.

## 4. Discussion

The integration of e-nose technology with TinyML for detecting diabetes mellitus through exhaled human breath has yielded promising results for an embedded system incorporating MOS sensors, aligning with prevailing research on diabetes mellitus detection and diagnosis via breath analysis [35,36,39,40]. However, the study faces several notable limitations and challenges.

Firstly, the sensitivity of the implemented MQ gas sensor series to high RH levels poses a significant challenge, necessitating continuous validation and calibration of the medical devices to detect errors in the system’s operation promptly. This is crucial, especially considering the limited lifespan and susceptibility of MOS sensors to %RH [16,24,35,45,46,48,49]. Additionally, the need for a dehumidifier to clean the e-nose sample chamber adds complexity to sensor maintenance and operational logistics. Moreover, the lack of a standardized protocol for employing Tedlar bags in exhaled-breath collection limits generalizability across studies focusing on detecting biomarkers of other diseases. Furthermore, issues such as sensor sensitivity, drift, and inaccuracies in breath sampling compound the study’s limitations. These challenges can introduce variability and inaccuracies in the data collected, potentially affecting the performance and reliability of the developed models. The selection of sensors depends on their implementation in the algorithm and the intricacy of feature extraction and selection using computational techniques like PCA and SVD [35,40]. However, these techniques may introduce complexity to the classification routine, impacting microcontroller speed and processing capabilities, particularly with arrays exceeding 10 gas sensors in the e-nose [48].

Furthermore, the study’s reliance solely on deep learning models for embedded system implementation presents a significant constraint. While deep learning algorithms show promise, the absence of alternative machine learning libraries compatible with TinyML integration restricts model diversity. The most suitable algorithms, DNN and 1D-CNN, were determined based on scoring metrics and false-positive or -negative avoidance. These algorithms have proven effective in diabetes mellitus detection, as corroborated in previous studies in cloud-based systems or processing results on a computer [44,51,52]. While effective, limitations persist, notably with TinyML integration and the explainability of certain algorithms, hindering diagnosis interpretation in case of misdiagnosis [53,54,55,56]. In contrast, the XGBoost algorithm can be converted to these formats but with the disadvantage of losses in precision and recall metrics, making the process more complicated. Not all algorithms are explainable, making it practically impossible to discover the factors influencing the model’s decision in case of a misdiagnosis [16,35]. Consequently, the potential for obtaining quantitative BGL predictions is hindered, highlighting the need for further development in this area [49].

Moreover, the reliance on a small sample size for breath tests limits the generalizability and robustness of our findings. While distinct BGL patterns were discernible among different patient groups within this dataset [44,46,49], expanding the participant pool to encompass a broader spectrum of individuals with diverse medical histories, treatments, and lifestyles is imperative [16,35,51,52]. Such an expansion would not only enhance system generalization and performance but also render it a more precise and robust embedded system for breath analysis, providing a valuable qualitative tool for medical practitioners.

Additionally, the accuracy assessment of our algorithm was confined to tests conducted solely on the included participants. Thus, broadening the participant pool to account for individual parameter variability becomes necessary to effectively train models. This comprehensive approach would bolster generalization and performance, culminating in a more precise and robust embedded system for breath analysis across diverse patient cohorts. Ultimately, such advancements would provide invaluable support for medical professionals in their diagnostic and monitoring endeavors [35,55,59].

The integration of TinyML into e-nose reliability remains uncertain, particularly in healthcare contexts where patient well-being is paramount. Aging device components can introduce variations in battery life, accuracy, and data collection by sensors, further emphasizing the importance of preprocessing, sensor types, and their number in influencing model accuracy and maximum memory.

While this study demonstrates promise in applying e-nose technology integrated with TinyML for diabetes mellitus detection, it is crucial to address the aforementioned limitations. Future research efforts should focus on overcoming these constraints to bolster the reliability and applicability of embedded systems for noninvasive diabetes monitoring.

## 5. Conclusions

This study underscored the promising application of integrating an e-nose with TinyML for noninvasive diabetes detection through human breath analysis. The implementation of algorithms such as XGBoost, DNN, and 1D-CNN yielded significant results, achieving accuracies of 91% and 94.4% in classification, respectively. These findings highlight the feasibility of this technology for the qualitative classification of BGLs, distinguishing between low BGLs for the HI group or high levels for the DMI group.

While XGBoost has emerged as the most suitable algorithm for this task [39,51,55], challenges were encountered in its conversion and compatibility with TensorFlow Lite, particularly when compared to other algorithms like SVM, KNN, and Random Forest. Nonetheless, DNN and 1D-CNN have proven effective, demonstrating favorable metrics in terms of precision and recall, showcasing their applicability in embedded systems [56,57,58].

The discussion emphasizes the importance of expanding the dataset to include a more diverse range of patients to enhance the system’s robustness. Additionally, addressing technical limitations, such as developing standardized protocols for breath sample collection and optimizing the integration of TinyML with machine learning algorithms, is crucial to improve accuracy and interpretability [16,39,60,61,62].

Furthermore, it is essential to recognize the potential clinical impact and practical relevance of the study’s findings. The successful implementation of integrated systems for diabetes mellitus detection could enhance early diagnosis, disease management, and patient quality of life [4,8,16,23,24]. Emphasizing interdisciplinary collaboration among data scientists, biomedical engineers, clinicians, and other healthcare professionals is vital to advance the development and implementation of innovative technologies like the combination of e-nose and TinyML in medicine [53,55]. Such collaboration fosters a holistic approach to healthcare innovation, facilitating the translation of research findings into practical solutions that benefit patients and healthcare providers alike.

Additionally, future research efforts should prioritize optimizing sensor selection, improving algorithm interpretability, and validating system performance across a broader range of populations and clinical conditions [8,9]. Addressing technical and methodological limitations identified in this study will not only enhance the reliability and applicability of the developed system but also contribute to advancing the field of noninvasive diabetes monitoring and diagnosis [16,35,36,37,38].

Despite the challenges encountered, the integration of TinyML into Arduino Nano 33 BLE Sense represents a significant step toward the practical implementation of these models in embedded devices. Continuous validation and calibration are necessary to ensure precise system operation over time, particularly considering the limited lifespan of MOS sensors [16,24,39]. This study provides valuable insights into the application of TinyML in diabetes detection through human breath analysis. Although technical challenges persist, the results support the feasibility of implementing embedded systems for efficient and noninvasive monitoring of BGL, offering exciting opportunities for the future development of innovative medical devices.

## 6. Patents

A Utility Model application has been submitted to the Mexican Institute of Industrial Property (IMPI), which has successfully passed the formal examination by meeting the requirements established by the Federal Law on Industrial Property and the Regulations of the Industrial Property Law in Mexico. The Utility Model has been published in the IMPI database called SIGA 2.0 since 15 February 2024, identified with application number MX/u/2023/000465.

## Figures and Tables

**Table 1:** Sensors used in proposed e-nose system.

Sensor	Target Gases	Detection Range of Target Gas	Sensor Materials	Environment Condition Working
MQ–2	$H_{2}$, LPG, ${\mathrm{CH}}_{4}$, CO, alcohol, propane, air	200–10,000 ppm CO	Gas-sensing layer: ${\mathrm{SnO}}_{2}$ Electrode: Au Electrode line: Pt Heater coil: Ni-Cr alloy Tubular ceramic: ${\mathrm{Al}}_{2}O_{3}$ Anti-explosion network: stainless steel gauze (SUS316 100-mesh) Clamp ring: copper-plated Ni Resin base: Bakelite Tuber pin: copper-plated Ni	Temperature: −10–50 °C RH: less than 95% Standard detecting condition: 20 °C ± 2 °C temperature, 65% ± 5% humidity
MQ–3	Alcohol, benzine, ${\mathrm{CH}}_{4}$, hexane, LGP, CO, air	0.1–10 mg/L alcohol		
MQ–7	$H_{2}$, CO, LPG, ${\mathrm{CH}}_{4}$, alcohol, air	50–4000 ppm CO		
MQ–135	${\mathrm{CO}}_{2}$, alcohol, air, ${\mathrm{NH}}_{4}$, toluene, acetone, CO	0–200 ppm acetone		
MQ–138	Benzene, CO, ${\mathrm{CH}}_{4}$, n-hexane, alcohol, propane, air	200–10,000 ppm benzene		
DHT–22	Temperature, relative humidity	−40 °C–80 °C temperature, 0–100% relative humidity	Humidity-sensitive capacitive element: polymer material with high dielectric constant Thermistor: ceramic semiconductor material Housing: durable plastic or resin Electrical connectors: copper or gold-plated metals	Temperature: 0–50 °C RH: 0–100%
MICS–5524	CO, VOCs, $C_{2}H_{6}$OH, $H_{2}$, ${\mathrm{NH}}_{3}$, ${\mathrm{CH}}_{4}$	1–1000 ppm VOCs	Catalytic detection electrode: Pt Counter electrode: Pt Ceramic components: fabrication of the sensor substrate Metallic components: steel wires or other metallic materials used in the construction of the sensor encapsulation	Temperature: 23 °C ± 5 °C RH: less than 95%

**Table 2:** Physical information of 44 participants.

Parameter	Healthy Patients	Diabetes Mellitus Patients
Age (yr.)	23.64 ± 2.19	29.95 ± 4.24
Height (cm)	1.72 ± 0.12	1.69 ± 0.12
Weight (kg)	72.16 ± 10.38	76.80 ± 11.42
BMI (kg/m^2^)	24.47 ± 4.02	27.33 ± 6.20
Gender (M/F)	13 M/9 F	10 M/12 F
Number of participants by healthy individuals (HI) or type of diabetes mellitus (T1DMI/T2DMI)	22 HI	8 T1DMI/14 T2DMI
Minimum and maximum BGL (mg/dL)	80.59/94.63	139.28/303.10

**Table 3:** Optimal hyperparameters for XGBoost using grid search and cross-validation.

Parameter	Value
Learning rate	0.05
Max depth	3
N estimators	100
Regularizes (alpha and lambda)	1, 0.1

**Table 4:** XGBoost algorithm classification report.

Detection	Precision	Recall	F1-Score	Support
HI	0.91	1.0	0.95	10
DMI	1.00	0.90	0.95	10

**Table 5:** DNN and 1D-CNN algorithm classification report.

Detection	Precision	Recall	F1-Score	Support
HI	0.90	1.0	0.95	9
DMI	1.00	0.89	0.94	9

## Data Availability

The data presented in this study are available on request from the corresponding author.
